# A New Pathway for Senescence Regulation

**DOI:** 10.1016/j.gpb.2015.11.002

**Published:** 2016-01-08

**Authors:** Xi Cao, Mo Li

**Affiliations:** 1 School of Basic Medical Sciences, Peking University Health Science Center, Beijing 100191, China; 2 Center for Reproductive Medicine, Peking University Third Hospital, Beijing 100191, China

Research concerning senescence has become a hotspot since the conception of ‘cellular senescence’ was put forward by Drs. Hayflick and Moorhead over five decades ago [1]. Recently, a paper published in *Science* by Kang and colleagues, which this article aims to comment on, provides evidence of a new pathway involved in senescence [2]. Senescence is a physiological and pathological process induced by numerous factors, during which cell growth ceases and gene expression alters. As can be imagined, such state protects against the development of cancer and plays an important role in tissue repair [3]. On the other hand, senescence is involved in many other processes such as aging and neurodegenerative disease [4], [5], [6]. Recent studies show that senescent cells produce senescence-associated secretory phenotype (SASP) factors including chemokines, proteases, pro-inflammatory cytokines, growth factors, macrophage inflammatory proteins (MIPs), and granulocyte–macrophage colony-stimulating factors (GM-CSFs) [7], [8]. It is known that SASP is mediated by the transcription factors NF-κB [9]. But how SASP is initiated and maintained is not clear.

There are up to 10^6^ DNA damages happening in each cell per day. To respond to these threats, cells evolve the DNA damage response (DDR) system to sense and repair the damages if these damages are not so severe. However, if the damages are beyond the capability of the cell to repair, DDR will direct other ways to maintain the genomic stability, such as apoptosis and senescence [10]. In other words, upon the stimuli such as ionizing radiation, genotoxic drugs, or replication errors, cells may undergo senescence [11].

GATA4, a GATA family member, is a transcriptional regulator, which possesses a zinc-finger domain. GATA4 is well recognized for its involvement in the regulation of embryonic development of heart, testis, ovary, ventral pancreas [12], [13]. In the work by Kang et al. it is shown for the first time that GATA4 plays a critical role in mediating senescence [2].

Traditionally it is thought that DDR induces cellular senescence primarily through two pathways. One pathway requires the mediation by p53. When receiving a signal from DDR, p53 is activated and induces the expression of p21, a cyclin-dependent kinase (CDK) inhibitor, arresting the progress of the cell cycle [14], [15]. The other is p16-retinoblastoma (pRB) pathway, which works by inducing the expression of p16, another CDK inhibitor. p16 keeps pRB in an active state and blocks cell proliferation by suppressing E2F, a transcription factor regulating cell cycle, thus leading to growth arrest [15]. Both pathways ultimately act on cell cycle and achieve growth arrest [16].

In their paper, Kang et al. establish a new DDR-inducing senescence pathway, in which GATA4 mediates cellular senescence, not by inhibiting cell cycle but by regulating SASP through NF-κB. In this pathway, GATA4 is regulated by autophagy, rather than, generally thought, by protease [2]. The experiments were well organized and elaborately designed. The authors demonstrate GATA4 as a novel senescence regulator by evaluating the influence of ectopic expression of GATA4 in normal cells. Since proteins are degraded by either the ubiquitin–proteasome pathway or autophagy-lysosome pathway in eukaryotic cells, the cells were treated with inhibitors of these two pathways, respectively. As a result, GATA4 was found to be regulated through the autophagy pathway. Then, the authors designed a set of experiments to figure out the upstream regulators and downstream effectors of GATA4 and finally established a new branch of senescence regulatory pathway [2]. In this GATA4 pathway, DDR is the initiator of senescence process, which activates the two key kinases, ataxia telangiectasia mutated (ATM) and ataxia telangiectasia and Rad3-related (ATR) [17]. Both ATM and ATR inhibit p62, an autophagy adaptor responsible for selective autophagy of GATA4, resulting in the increase of GATA4. NF-κB is then activated through tumor necrosis factor receptor-associated factor interacting protein 2 (TRAF3IP2) and interleukin 1A (IL1A), finally inducing the secretion of SASP factors and leading to senescence [2] (Figure 1).

The authors went further and put forward an idea to patch up the historic argument concerning whether autophagy has positive or negative effect on senescence [18]. Some previous studies suggest that autophagy is important for the establishment of senescence, while others are indicative of the protection effect of autophagy against senescence [19], [20]. In the present work, autophagy is divided into two types, selective autophagy and general autophagy. While selective autophagy decreases the expression of senescence-positive factors such as GATA4, general autophagy results in senescence. When given senescence-inducing stimuli, selective autophagy is firstly initiated, protecting cells against senescence. Subsequently, general autophagy starts, leading to cellular senescence [2].

It’s obvious that this study is of great importance both in scientific research and in clinical practice. For example, from now on, GATA4 will be considered not only a transcription factor involved in embryonic development, but also an essential component in senescence regulation. Also, it’s not hard to imagine that a new model for senescence aiming at GATA4 pathway could be established in the near future. As for clinical trials, novel targets for drug design and development are going to emerge, indicative of new therapeutic methods. No doubt, this work could provide insight into the intervention and treatment of cancer, premature senescence, neurodegeneration and aging-related diseases.

## Competing interests

The authors declare no competing interests.

## Figures and Tables

**Figure 1:**
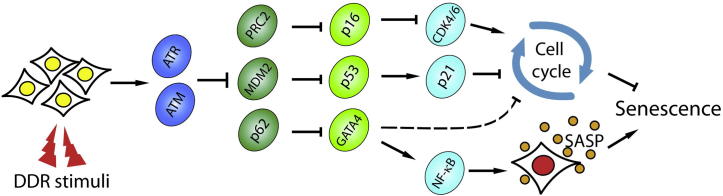
**Pathways of DDR-induced senescence** Upon the signal from DDR, the two upstream kinases, ATM and ATR, are activated, initiating the senescence. Classical senescence routes consist of the p53 pathway and p16-pRB pathway, which act on arresting cell cycle. The third one is mediated by GATA4, which functions by regulating SASP, finally resulting in senescence. DDR, DNA damage response; ATM, ataxia telangiectasia mutated; ATR, ataxia telangiectasia and Rad3-related; pRB, retinoblastoma; SASP, senescence-associated secretory phenotype; PRC2, polycomb repressive complex 2; MDM2, mouse double minute 2 homolog.
